# A Comparative Effectiveness Study of Newborn Screening Methods for Four Lysosomal Storage Disorders †

**DOI:** 10.3390/ijns6020044

**Published:** 2020-05-30

**Authors:** Karen A. Sanders, Dimitar K. Gavrilov, Devin Oglesbee, Kimiyo M. Raymond, Silvia Tortorelli, John J. Hopwood, Fred Lorey, Ramanath Majumdar, Charles A. Kroll, Amber M. McDonald, Jean M. Lacey, Coleman T. Turgeon, Justin N. Tucker, Hao Tang, Robert Currier, Grazia Isaya, Piero Rinaldo, Dietrich Matern

**Affiliations:** 1 Biochemical Genetics Laboratory, Department of Laboratory Medicine and Pathology, Mayo Clinic, Rochester, MN 55905, USA; sanders.karen@mayo.edu (K.A.S.); gavrilov.dimitar@mayo.edu (D.K.G.); oglesbee.devin@mayo.edu (D.O.); raymond.kimiyo@mayo.edu (K.M.R.); tortorelli.silvia@mayo.edu (S.T.); Majumdar.Ramanath@mayo.edu (R.M.); kroll.charles@mayo.edu (C.A.K.); mcdonald.amberm@mayo.edu (A.M.M.); lacey.jean@mayo.edu (J.M.L.); turgeon.coleman@mayo.edu (C.T.T.); rinaldo@mayo.edu (P.R.); 2 Department of Clinical Genomics, Mayo Clinic, Rochester, MN 55905, USA; 3 Lysosomal Diseases Research Unit, South Australian Health and Medical Research Institute, Adelaide 5000, Australia; john.hopwood@sahmri.com (J.J.H.); jntucker@gmail.com (J.N.T.); 4 Genetic Disease Screening Program, California Department of Public Health, Richmond, CA 94804, USA; fred_lorey@sbcglobal.net (F.L.); hao.tang@cdph.ca.gov (H.T.); robert.currier@ucsf.edu (R.C.); 5 Department of Pediatrics, University of California, San Francisco, CA 94143, USA; 6 Department of Pediatric and Adolescent Medicine, Mayo Clinic, Rochester, MN 55905, USA; gisaya01@gmail.com

**Keywords:** newborn screening, Fabry disease, Gaucher disease, mucopolysaccharidosis type I, Pompe disease, tandem mass spectrometry, immunoassay, microfluidics, bioinformatics, post-analytical interpretation

## Abstract

Newborn screening for one or more lysosomal disorders has been implemented in several US states, Japan and Taiwan by multiplexed enzyme assays using either tandem mass spectrometry or digital microfluidics. Another multiplex assay making use of immunocapture technology has also been proposed. To investigate the potential variability in performance of these analytical approaches, we implemented three high-throughput screening assays for the simultaneous screening for four lysosomal disorders: Fabry disease, Gaucher disease, mucopolysaccharidosis type I, and Pompe disease. These assays were tested in a prospective comparative effectiveness study using nearly 100,000 residual newborn dried blood spot specimens. In addition, 2nd tier enzyme assays and confirmatory molecular genetic testing were employed. Post-analytical interpretive tools were created using the software Collaborative Laboratory Integrated Reports (CLIR) to determine its ability to improve the performance of each assay vs. the traditional result interpretation based on analyte-specific reference ranges and cutoffs. This study showed that all three platforms have high sensitivity, and the application of CLIR tools markedly improves the performance of each platform while reducing the need for 2nd tier testing by 66% to 95%. Moreover, the addition of disease-specific biochemical 2nd tier tests ensures the lowest false positive rates and the highest positive predictive values for any platform.

## 1. Introduction

With the availability of more treatment options and population screening assays for an increasing number of lysosomal storage disorders (LSDs), the inclusion of these conditions into newborn screening programs seems reasonable because existing evidence suggests that early treatment initiation has the greatest benefit. Indeed, Pompe disease (due to a deficiency of acid alpha-1,4-glucosidase, GAA) and mucopolysaccharidosis type I (MPS I, due to a deficiency of alpha-L-iduronidase, IDUA) have already been added to the US Secretary of Health and Human Services’ Recommended Uniform Screening Panel (RUSP) [1]. In Taiwan, newborn screening for Pompe disease and Fabry disease (due to a deficiency of alpha-galactosidase A, GLA) has been ongoing since 2005 and 2006, respectively [2,3]. In addition, a few states in the US have begun to screen for up to seven LSDs using one of several laboratory methods that have been proposed for this purpose. At present, most programs required to screen for one or more LSD make use of enzyme activity assays performed on extracts from newborn screening dried blood spots (DBS) and the subsequent measurement of enzyme product by tandem mass spectrometry (MS/MS) [4,5,6,7]. At least one state program employs a fluorometric approach on a digital microfluidics (DMF) platform [8]. Both of these options allow for the determination of multiple enzyme activities in a single analysis, which is efficient in terms of sample amount, equipment and reagent needs, as well as labor. A similar multiplex approach does not measure enzyme activities, but enzyme concentration, as an indicator for disease, because in most LSDs the pathogenic mutations lead to a decreased amount of protein with subsequent abnormally low enzyme activity [9,10]. This assay applies microbead array technology for the immune quantification of lysosomal proteins in DBS and requires protein-specific antibodies that—in contrast to the reagents needed for the MS/MS and DMF assays—are currently not commercially available. To screen for a subgroup of LSDs, the mucopolysaccharidoses (including MPS I), the measurement of glycosaminoglycans in DBS has also been suggested, but this approach cannot be limited to a single condition, such as MPS I, and it has not yet been applied in a prospective screening study [11]. 

To determine which analytical method is the most efficient and effective newborn screening procedure, we assessed their performance in a comparative effectiveness study of nearly 100,000 de-identified residual newborn screening specimens. The MS/MS based assay can be multiplexed to include at least six lysosomal enzymes, as well as four lysophosphatidylcholines, to screen for X-adrenoleukodystrophy (ALD) [6]; and the immunocapture assay can be multiplexed for at least 13 lysosomal proteins [10,12], frataxin (a marker for Friedreich ataxia), and ceruloplasmin (a marker for Wilson disease, Menkes disease and aceruloplasminemia) [13]. The DMF-based assay can measure up to five enzyme activities simultaneously [14], but at the time of our study it was limited to four [8]. The focus of this report is the four LSDs that can be screened for by all three of the multiplex methods, specifically Fabry disease, Gaucher disease (due to deficiency of acid beta-glucosidase, GBA), MPS I, and Pompe disease.

## 2. Materials and Methods 

### 2.1. Study Population

A comparative effectiveness study of 100,000 newborn screening specimens was conducted in order to determine the most efficient and effective approach to newborn screening for Fabry disease, Gaucher disease, MPS I, and Pompe disease. The newborn screening specimens were obtained over a period of two years from the Genetic Disease Screening Program of the California Department of Public Health (CDPH). These residual specimens were from routine newborn screening and were sent to Mayo Clinic once all state mandated testing was completed by the CDPH, on average six weeks after sample collection. Specimens were kept frozen following testing at CDPH until testing at Mayo Clinic. All remaining specimens were returned to CDPH.

Between August 2011 and September 2013, the CDPH submitted every week approximately 1000 residual newborn screening specimens to the Mayo Clinic’s Biochemical Genetics Laboratory. All patient identifiers were removed prior to shipment and the following demographic information, as disclosed on the screening specimen card, was shared with Mayo Clinic: sex, birth weight (rounded to nearest 100 g increment), gestational age, ethnic background, and age at specimen collection (in hours). While only 10% of the California newborn population during the two years (500,000 live births per year) was the subject of this study, CDPH made every effort to submit specimens with an equal sex distribution and reflecting the diversity of the California population in terms of gestational age and ethnic background, as disclosed with every DBS specimen. Mayo Clinic’s institutional review board (IRB #10-005821) and the California Health and Human Services Agency’s Committee for the Protection of Human Subjects reviewed the study protocol and deemed the study not human subject research, as defined in 45 CFR 46.102(f)(2), because no identifiable specimens of newborn blood spots were to be released by the CDPH to Mayo Clinic.

### 2.2. Study Protocol

Mayo Clinic’s Biochemical Genetics Laboratory implemented three high-throughput screening assays, each designed for the simultaneous measurement of multiple biomarkers in DBS specimens for the detection of at least four LSDs, specifically Fabry disease, Gaucher disease, MPS I, and Pompe disease [6,8,10]. The initial reference ranges and cutoffs for each enzyme activity or concentration level were determined following the analysis of at least 120 residual newborn screening specimens and available specimens from affected patients. The protocol called for the analysis of the weekly shipments of study specimens using the three screening tests in daily batches of 200 to 250 specimens. Because all specimens were de-identified, the clinical follow up of abnormal results was not possible. To confirm a presumptive diagnosis, supported by at least two of the three screening tests, additional biochemical, and ultimately molecular genetic analysis of relevant genes, was to be performed. Repeat testing by the same method to confirm an out-of-range result was done initially but abandoned so not to waste the specimens, given the availability of results for the same condition from the other tests. Previously described fluorometric standalone enzyme assays [15,16,17,18] were implemented to serve as 2nd tier biochemical genetic tests. A normal 2nd tier enzyme assay was to overrule the primary screening result, which would then be considered false positive. If the results of a 2nd tier test supported the finding of the primary screening tests, the final diagnostic confirmation was to be accomplished by molecular genetic analysis using clinically available Sanger sequencing assays. A graphical description of the study was previously published [13]. Since the completion of the prospective study in 2013, several additional DBS-based biomarker assays were implemented: dermatan and heparan sulfates for MPS I [19], glucosylsphingosine for Gaucher disease [20], and creatine and creatinine for Pompe disease [21]. These biomarkers were measured in any available leftover study specimens that had tested presumptively positive during the course of the study and revealed genotypes of variable significance. Because globotriaosylsphingosine, the only biomarker for Fabry disease in blood, is not reliably elevated, in particular in newborns with Fabry disease [22], the determination of disease state in screen-positive cases was based upon molecular genetic testing of *GLA*. Gene variants were interpreted according to current guidelines established by the American College of Medical Genetics and Genomics (ACMG) [23].

### 2.3. Statistical Analysis

The initial decision of normal vs. presumptive positive screening results was based on cutoffs determined by the comparison of the reference range for each of the four enzymes, tested by each of the three screening tests in at least 120 random residual newborn screening DBS, to the results obtained in specimens from patients with Gaucher disease (*n* = 53), Fabry disease (*n* = 39 male patients), MPS I (*n* = 8), and Pompe disease (*n* = 24). The DBS of these patients were created from leftover whole blood submitted for diagnostic enzyme analysis and/or molecular genetic testing. Accordingly, these specimens are not equivalent to DBS collected for newborn screening. The cutoffs were set to minimize the possibility of a false negative result. Performance metrics were calculated for each primary screening test based on the respective results and the final determination of true or false positive outcomes, based on the laboratory data obtained. The metrics included sensitivity, specificity, false positive and negative rates, as well as positive and negative predictive values. The detection rate was determined for each assay and condition but was not included in the calculation of the performance metrics. 

Upon the completion of the laboratory work, a web-based application, Collaborative Laboratory Integrated Reports (CLIR, version 2.18, https://clir.mayo.edu/) was created to determine if this multivariate pattern recognition software could improve the specificity of each screening test and reduce the need for 2nd tier testing while maintaining the highest possible sensitivity. CLIR is a custom-designed and coded application for the processing of laboratory data based on numerical results [24,25,26]. The development of its original version [27], as part of the Region 4 Collaborative project for MS/MS data sharing and comparison, was prompted after amino acid and acylcarnitine analyses by MS/MS were implemented in newborn screening programs, which had caused a significant rise in false positive results due to the complexity of interpreting metabolite profiles that are made up of more than 50 analytes [28,29]. CLIR is not based on the application of traditional analyte-specific cutoffs established after the analysis of a set of normal control specimens. Instead, it allows for the establishment of disease and control profiles against which every new case is compared and a likelihood of disease score is determined based on the degree of overlap of each case’s profile with condition-specific disease and control ranges. The use of CLIR in newborn screening has been shown to significantly reduce false positive results and improve the positive predictive value while maintaining, if not improving, sensitivity [25]. CLIR was applied to this study given its added ability to incorporate in its algorithm non-analytical variables such as sex, birth weight, gestational age, and age at the time of specimen collection.

The impact of available 2nd tier tests on screening performance and efficiency was also evaluated as an additional means to gain efficiency when screening was based either on traditional cutoffs or when using CLIR.

## 3. Results

### 3.1. Participants

Nearly 100,000 newborn DBS were screened for Fabry disease, Gaucher disease, MPS I, and Pompe disease by the immunocapture (*n* = 99,798), the MS/MS (*n* = 99,627), and the DMF assay (*n* = 90,498). The difference in numbers was due to individual assay failures not repeated when the other two assays were normal, and the fact that the DMF platform was not available until six weeks after the study had begun. There was a sufficient amount of specimens for a total of 89,508 newborns to be tested with all three methods and this set was considered the study population. Complete information about sex, birth weight, and age at collection was available for 97.4%, 97.6%, and 97.8% of participants, respectively. 

The DBS were randomly selected from California-wide residual specimens and the comparison of the ethnic background of neonates as recorded on each newborn screening card to the corresponding birth data for California [30] confirmed that a similarly diverse population sampling was achieved (Table 1). Of the study population, 47.5% were female and 49.9% were male; sex was not available for the remaining 2321 newborns. The median birth weight was 3300 g (mean: 3307 g; range: 300 to 9900 g). The median age at sample collection was 27 h; 114 cases were older than 4 weeks (mean: 36 h; range: 1 h to 367 days).

### 3.2. Cutoff Determination and Assay Performance

**Figure 1:**
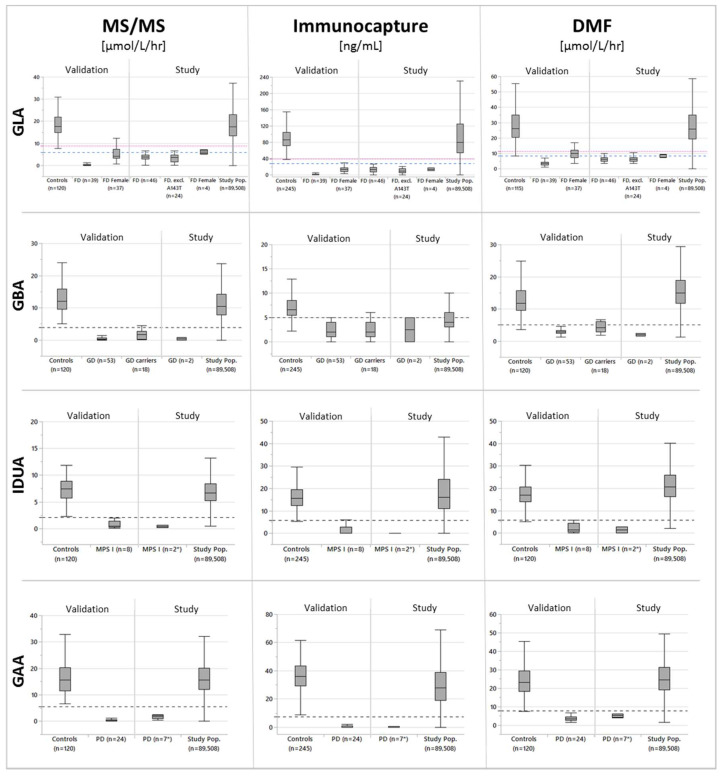
Validation and study data for each of the three screening platforms and enzymes represented as quartile plots (upper whisker end: 1.5 × interquartile range; top of box: 75th percentile; line in box: median; bottom of box: 25th percentile; lower whisker end: −1.5 × interquartile range). The horizontal lines indicate the chosen cutoffs during assay validation (Table 2). GLA cutoffs for females and males are provided in red and blue, respectively. FD, Fabry disease; GD, Gaucher disease; MPS I, mucopolysaccharidosis type I; PD, Pompe disease. * Includes one specimen from a known patient with PD and MPS I that was added randomly to the study population to test the systems.

Cutoffs for each enzyme and platform were established following the analysis of at least 120 residual newborn screening samples and comparison to the respective ranges observed in the available specimens of patients affected with the relevant disorder. The goal was to minimize the possibility of a false negative result. When there was separation between reference and disease range, the cutoff was determined as the mean of the interval between the lowest result of the reference range and the highest result of the disease range. However, because the complete separation of control and disease ranges was not achieved for each enzyme activity and each platform, the cutoff was set at the 99th percentile of the disease range, as long as at least one other platform showed separation. The rationale was that an affected newborn would be identified by at least one assay while limiting the need for 2nd tier testing. This was possible for GAA, GBA, and IDUA, however, for GLA only when targeting the positive identification of males with Fabry disease while accepting the inability to identify all female GLA mutation carriers (Figure 1).

Table 2 lists the cutoffs and the resulting number of specimens yielding abnormal results for each 1st tier testing platform. The immunocapture approach had the worst performance in this comparison, particularly for GBA, for which the results were below the cutoff in half of all cases which, however, was consistent with previous observations [10]. Therefore, the decision was made to ignore isolated low GBA concentrations and rely on enzyme assays to determine if additional testing should be done. The performance for the other assays was better, albeit none reached the acceptable performance metrics by themselves. However, the respective 2nd tier fluorometric single enzyme assays reduced the number of false positive results to more appropriate levels [31]. Genotypes for the respective genes (GAA, GBA, GLA, and IDUA) were determined to adjudicate the designation as true or false positive. This was done for those specimens with abnormal 2nd tier test results, but also for specimens for which at least two of the three 1st tier assays yielded abnormal results, or one 1st tier assay result was abnormal while the other two results were within 1.0 unit above their respective cutoff, even when the 2nd tier enzyme assay result was normal, or when insufficient specimen was left over to allow the performance of both 2nd tier and molecular genetic testing. This approach was taken by the primary data reviewer (D.M.) as a precautionary measure to limit the possibility of false negative 2nd tier test results or the inability to perform genotyping because of a lack of specimen. Indeed, as Table 3 shows, many cases with abnormal GLA activities by the 1st tier tests had normal activities by the 2nd tier fluorometic enzyme assay, in particular when the c.352C > T (p.Arg118Cys) variant was present. This variant is interpreted as likely to be pathogenic by some and of uncertain significance by others [32]. Because we identified this variant in eight males with low GLA activities in two or more 1st tier assays (except for the very first case, which was abnormal for the immunocapture assay only), in the end, we categorized this variant as of uncertain clinical significance.

As is also shown in Table 3, the molecular genetic analysis was inconclusive in another nine of 36 cases because they were carriers of at least one variant of uncertain significance (excluding individuals with the GLA p.A143T variant). Therefore, an attempt was made to delineate the clinical relevance of several cases with such genotypes of uncertain significance with biomarker assays that have become available after the prospective phase of the study had ended. This was possible for Pompe disease (creatine and creatinine [21]), Gaucher disease (glucosylsphingosine [20]), and MPS I (dermatan and heparan sulfates [19]), and was conclusive for cases where the specimen was still retrievable. However, because these tests were not available until up to five years after the initial screening tests were performed, doubt remains as to whether the normal results for these biomarker tests can be considered reliable after such lengthy storage. Nevertheless, among the 89,508 newborns screened with all three 1st tier assays, six cases were identified with overall results consistent with Pompe disease (one in 14,918), one with MPS I, two with Gaucher disease (one in 44,754), and 24 males with Fabry disease (one in 1852 males). Another 22 male participants had biochemical results suggestive of Fabry disease but GLA sequencing revealed only the p.A143T mutation, the clinical significance of which remains undetermined [33]. In addition, four female participants were found to carry a GLA mutation, including one with the p.A143T variant. All three platforms revealed a few false negative results (DMF for Fabry and Pompe diseases, MS/MS for Fabry disease, and immunocapture for Gaucher disease; Table 3). Based on these outcomes, performance metrics were calculated for the two-tier-screening approach, including each of the three primary screening platforms and the respective fluorometric enzyme assay as a 2nd tier test. These data are presented in Table 4, Table 5, Table 6 and Table 7 and Tables S1–S4.

### 3.3. Performance When Using CLIR’s Postanalytical Multivariate Pattern Recognition Tools

The data set used for analysis with CLIR was limited to those cases for whom the covariates birth weight, age at collection (< 1 year of age), and sex were available and there were no obvious typographical errors. This population comprised 87,321 cases (2187 fewer than or 97.6% of the population with results for each 1st tier test). As shown in Table 4, Table 5, Table 6 and Table 7, the application of the postanalytical CLIR tools led to a significant reduction in the need for 2nd tier testing and an improvement of false positive rates and positive predictive values. However, it did not seem to improve sensitivity. While it may be desirable that individuals carrying the p.A143T variant in GLA are not detected, assuming the final diagnoses were correct, CLIR led to a decreased sensitivity in screening for Fabry disease by immunocapture and MS/MS, and for Pompe disease by immunocapture.

## 4. Discussion

Newborn screening for one to seven different LSDs has become a reality in the United States, Taiwan, and Japan. Two testing platforms are established. Both seek to identify patients through the measurement of enzyme activity and both make use of one 3 mm disc punched from each newborn dried blood specimen. Both can measure several enzyme activities simultaneously. One measures the relevant enzyme’s product by MS/MS after an enzyme reaction was allowed to occur, usually overnight, in a 96-well format [6]. The other uses DMF to allow the enzyme reactions to occur in a closed system that provides results for 42 samples within 4 h in 48-well single-use cartridges [8]. A third approach relies on the quantitation of enzyme protein in a 96-well format by fluorometry after overnight incubation with polyclonal antibodies derived in sheep in one of the authors’ (J.J.H.) laboratories [10].

During this study, each primary screening test resulted in a large number of positive results with the application and consideration of only the respective cutoffs that were initially selected based on a limited number of controls and specimens from affected patients (Table 2). However, it is important to note that the false positive rate for each 1st tier test likely would have been better and closer to the data shown in Table 4, Table 5, Table 6 and Table 7 and Tables S1–S4 if repeat testing had not been forgone for the lack of specimen. In routine newborn screening, especially for LSDs, most labs actually retest the same specimen in duplicate to achieve more reliable results and fewer false positives [8,34,35]. 

DMF scored best in terms of below cutoff rates (Table 2) but missed one case of Pompe disease and several with Fabry disease (Table 4 and Table 7, Tables S1–S4). The MS/MS-based enzyme assay’s below cutoff rates were closer to those of the DMF platform (Table 2), and had the fewest false negative results, all for Fabry disease (Table 4, Table S1). The immunocapture assay’s number of below cutoff results was the highest (Table 2), and still had one false negative result for a case with Gaucher disease (Table 5). To determine if a larger data set could have improved the cutoff-based approach, a retrospective comparison of the original control set to the study set of 89,508 newborn screening specimens indicated that the range of results of the large study population was generally broader and lower than the initial control range (Figure 1).

The experience of this study and those reported from several programs (Tables S1–S4) show that single marker screening assays may achieve the generally desired sensitivity of 100%, but they lack in specificity. Targets have been proposed for a combined false positive rate (≤0.3%) and positive predictive value (≥20%) for the application of amino acid and acylcarnitine analyses by MS/MS [31], which includes at least the 20 core amino acid, fatty acid, and organic acid disorders of the RUSP [1]. Based on those targets, the performance for single conditions must be significantly better to ensure the population and follow-up system are not burdened by the unnecessary follow-up of large numbers of false positive cases. Accordingly, better screening tests or screening strategies must be applied. For the four LSDs included in this study, a tiered approach seems best to achieve the goal of a well-performing newborn screening program. Our study, however, also shows that the fluorometric stand-alone enzyme assays are unreliable 2nd tier tests because they also lack sensitivity and specificity. This is consistent with reports from Taiwan where fluorometric enzyme assays for Pompe disease, Fabry disease, and MPS I had positive predictive values ranging from 0.37%–1.25% (Pompe [36,37]), 3.8%–11% (Fabry [3,38]), and 11% (MPS I [39]). Comparing the incidence of patients identified in our study to those reported by other programs indicates similar occurrences with at least one other screening program, albeit not for all diseases (Tables S1–S4). For Fabry disease, our findings correlate well with Missouri and Taiwan, but not New York and Illinois [7,34,40,41]. The frequency of Gaucher disease in the study population is identical to Illinois and close to Missouri. The observed difference to New York (mostly New York City) and Taiwan can be explained by the predominant ethnic background of the respective populations screened. One case of MPS I was identified, a higher incidence than in Missouri and Illinois, and lower than in Taiwan [42]. Most interesting is the comparison for Pompe disease because relevant data were recently published from CDPH indicating a frequency of one in 25,200 vs. one in 15,000 in our study [43]. While possibly coincidental, this difference raises the suspicion that some of the four different *GAA* variants of uncertain significance identified in this study may not cause disease. Indeed, our study shows that molecular genetic analysis is unable to conclusively determine disease status in many cases with below-cutoff enzyme activities and genotypes involving variants of uncertain significance. This is consistent with reports from New York and California, where genotyping has been used as a 2nd tier test for newborn screening for Krabbe disease [35,44] and Pompe disease, respectively [43]. Therefore, better biomarkers to serve as 2nd tier, if not primary screening, tests are essential. Such tests became available in recent years for Gaucher disease [20], MPS I [19], and Pompe disease [21], while a reliable marker for Fabry disease remains elusive. Because relevant tests for these markers came too late to be employed prospectively and consistently during this study, every effort was made to retrieve and test retrospectively any available leftover specimen that had tested presumptively positive per the study protocol. While it was not possible to measure glucosylsphingosine, dermatan and heparan sulfates, or creatine and creatinine for all cases at risk of Gaucher disease, MPS I, or Pompe disease, respectively, this helped to further clarify several presumptively positive cases as true or false. Moreover, a two-tier approach has meanwhile been shown to considerably improve the performance of newborn screening for Krabbe disease, MPS I, and Pompe disease [21,45,46].

Any 2nd tier test requires additional effort and the cost can be high when each abnormal 1st tier screening test triggers immediate 2nd tier analysis. However, because Gaucher disease, Fabry disease, and MPS I would not meet the criteria for time critical conditions [47], the 2nd tier tests do not need to be performed immediately, and samples can be batched for weekly analysis. Moreover, the regionalization of the 2nd tier tests is possible to further reduce the cost of these analyses without impacting appropriate patient care. Creatine and creatinine can even be measured simultaneously with amino acids and acylcarnitines. This allows for the timely reporting of results indicative of Pompe disease, particularly infantile onset Pompe disease, which is a time-critical condition for which the results should be reported by the 5th day of life and treatment be initiated before the 3rd week of life [48]). If guanidinoacetate was also measured, along with creatine and creatinine, then creatine deficiency disorders, such as GAMT deficiency, could be screened for as a secondary benefit to screening efficiently for Pompe disease [21,49].

To further improve the cost effectiveness of newborn screening for these four conditions, the data were interrogated by CLIR software, a suite of bioinformatics tools to rapidly sift through vast amounts of data in search for specific conditions. CLIR was first trained to recognize the four conditions using the control data set and the data from the known patients with the relevant diseases, as well as the cases proven to be healthy mutation carriers or having pseudodeficient enzyme activities. CLIR integrates not only each platform’s four enzyme results and all possible permutations of ratios but also the available demographic information, including age at specimen collection, birth weight, and sex. The output of the CLIR tools is a likelihood score of disease compared against populations of controls and false positive cases, including mutation carriers (for autosomal recessive conditions) and individuals with pseudodeficiency. The application of postanalytical CLIR tools would have reduced the need for 2nd tier testing for the four LSDs by 95%, 77%, and 66% for the immunocapture, MS/MS, and DMF platforms, respectively (Table 4, Table 5, Table 6 and Table 7). In addition, it improved the false positive rate and positive predictive value for all conditions and platforms. In terms of sensitivity, CLIR tools made no difference for DMF, but may have caused two more false negative cases for Fabry disease by MS/MS, and for the immunocapture assay, resulted in an additional three missed cases of Fabry disease and one of Pompe disease. However, in reviewing the particular cases categorized as not affected by CLIR, all male Fabry disease cases and one Pompe disease case have genotypes involving variants of uncertain significance (Table 3). In the absence of clinical follow-up, these cases’ diagnoses cannot be considered final. CLIR also produced a negative result for one case of Gaucher disease by the immunocapture assay, which by itself was borderline normal (GD #1 of Table 3). Sanger sequencing of this case revealed homozygosity for a pathogenic mutation and the diagnosis was further confirmed by the retrospective finding of elevated glucosylsphingosine (130 nmol/mL; controls < 47 nmol/mL) [20]. Finally, CLIR is a dynamic system and its performance can be improved through two mechanisms. One is the continued addition of data sets from controls, but particularly from affected and false positive cases, in order to refine the disease and non-disease profiles. The other is the inclusion of additional markers measured in the same specimen. For example, while this report was limited to the enzyme activities/concentrations to detect the four LSDs common to all three 1st tier assays, the immunocapture assay and the MS/MS method both can measure additional markers simultaneously with little impact on workload. MS/MS is already employed to screen for six and seven LSDs in Tennessee and Illinois, respectively. In the Kentucky newborn screening program for Krabbe disease, MPS I, and Pompe disease, up to 10 markers are used successfully by CLIR to minimize the need for 2nd tier tests [45], and Hall et al. have shown that CLIR’s ability to appropriately categorize cases as true or false positives increases when six vs. two enzyme results are available [50]. Because Meikle et al. have already shown that protein-to-protein ratios enhance the accurate interpretation of case-specific results [10], and because this study shows the benefit that CLIR provides even when using results for only four markers, it is only reasonable to expect that the further inclusion of the results for the other 14 protein concentrations measured by the immunocapture method [13] would improve the overall performance of this platform.

While some competing reports and opinions have been published about whether either the MS/MS- or DMF-based lysosomal enzyme assays are superior [51,52,53,54,55], our study is the first, and so far only, to compare prospectively two or more testing platforms on the same set of specimens. Perhaps the results could have been more favorable for any of the assays used if it had been possible to follow the initial study protocol, which called for the use of residual specimens received by Mayo Clinic’s Biochemical Genetics Laboratory on a routine and daily basis from the Minnesota Department of Health, as part of a public/private partnership geared to a shared and cost-effective state newborn screening program. However, once federal funding was obtained, the Minnesota Department of Health had to rescind its agreement to the use of the residual specimens because of legal issues surrounding the use of such specimens for research [56]. This prompted the need to establish a contract with CDPH, which had agreed in principle to serve as a backup source of specimens when the original request for funding was submitted to the Eunice Kennedy Shriver National Institute of Child Health and Human Development (NICHD). Moreover, it became necessary to obtain additional funding for CDPH for the effort to retrieve and send 100,000 residual specimens over two years. While this caused a delay of one year, the benefit was that the DMF platform could be used on almost all the study specimens. However, this backup plan also increased the time between DBS collection and testing because CDPH could not send any DBS until all in-state testing had been completed. Another major setback for the study occurred when the planned three-year study period came to an end because the NICHD had to apply a new rule that precluded no-cost extensions. This meant that a third of the original funding became suddenly unavailable, and to complete the study, additional time needed to be invested to obtain new grant support. 

## 5. Conclusions

This unique comparative effectiveness study of three primary screening platforms showed that each platform by itself is incapable of ensuring a cost-effective screening program for the four LSDs. An unexpected finding was that the immunocapture assay had more false positive cases than anticipated, as it also did not prevent the unnecessary identification of mutation carriers and individuals with genotypes conferring pseudodeficient enzyme activities. To be fair, however, the immunocapture assay was designed to multiplex 14 lysosomal marker proteins in a single assay to detect up to 15 different LSDs and to utilize resultant protein–protein ratios to reduce diagnostic errors [10,12]. Because the necessary reagents for this assay are currently not commercially available, this leaves programs that decide to screen for LSDs with the choice between two platforms. The DMF platform is a proprietary FDA-approved system (Baebies, Inc., Durham, NC, USA) that requires little desk space, little specimen handling, and no overnight incubation. The MS/MS-based method is dependent on commercial reagents—also FDA-approved—from one vendor (PerkinElmer, Inc., Waltham, MA, USA), but in contrast to DMF, can be modified by any laboratory to include more enzyme assays or other markers in a single assay. For example, Dr. Michael Gelb—who pioneered the MS/MS-based lysosomal enzyme assays for population screening [4]—already proposed multiplexing the enzyme assays with additional markers, such as lysosphingomylin for Niemann–Pick A and B diseases, that are currently used as 2nd tier tests [57]. Laboratories also need to consider each platform’s space and other infrastructure needs (e.g., ventilation, temperature stability, etc.) to ensure the timely screening of their populations. The eventual decision should be transparent and include a declaration of the goals of newborn screening for a particular condition. In the context of the RUSP, this means that it should be stated if all disease variants are to be identified or if the early onset phenotypes represent the core condition, while milder, later onset variants are secondary targets that may not be identified in all cases to ensure an acceptable false positive rate for the core condition. 

## Figures and Tables

**Table 1:** Racial/ethnic background of the study population in comparison to population data for neonates born during the same time frame (2011–2013) [30].

Race/Ethnicity	Study Population	California Birth Data *
White	37.8%	27.6%
Hispanic/Latina	41.6%	48.8%
African American/ Black	5.4%	5.3%
Asian/Pacific Islander	11.0%	13.6%
American Indian/ Alaska Native	0.14%	0.4%
Multiracial	-	2.2%
Other/Unknown	4.2%	2.2%

* Average of data from 2011, 2012, and 2013.

**Table 2:** First tier test cutoffs and number of cases below the cutoffs among the study population of 89,508 newborns (see Figure 1).

Enzyme	DMF [μmol/L/h]	Immunocapture [ng/mL]	MS/MS [μmol/L/h]
GAA	7.71 (*n* = 412, 0.412%)	7.36 (*n* = 4,478; 5.003%)	5.55 (*n* = 900, 1.005%)
GBA	5.02 ^1^ (*n* = 376, 0.420%)	5.00 ^1^ (*n* = 45,105; 50.392%)	3.95 (*n* = 1521; 1.699%)
IDUA	5.72 ^1^ (*n* = 268, 0.299%)	5.79 ^1^ (*n* = 4372; 4.884%)	2.1 (*n* = 312, 0.349%)
GLA ^2^	11.4 ^3^ (*n* = 2357, 2.633%)	39.36 ^3^ (*n* = 9764; 10.909%)	8.90 ^3^ (*n* = 3637; 4.063%)
GLA (males only) ^4^	8.36 (*n* = 473, 0.528%)	27.41 (*n* = 2931; 3.275%)	5.95 (*n* = 354; 0.395%)

^1^ Cutoffs represent the 99th percentile of the affected range; ^2^ data based on cutoffs targeting all male Fabry patients and most female GLA mutation carriers; ^3^ represents 1st percentile of control range; ^4^ data based on cutoffs targeting male patients with Fabry disease.

**Table 3:** Biochemical and molecular genetic results for the true positive cases identified during this study. Cutoffs for DMF, immunocapture and MS/MS assays are provided in Table 1. Values in bold face are below the relevant cutoffs.

Case	Enzyme/ Gene	DMF [µmol/L/h]	Immunocapture [ng/mL]	MS/MS [µmol/L/h]	2TT ^1^ [µmol/L/h]	Genotype Interpretation ^2^	Variant 1	Variant 2
FD #1	GLA	9.1 *	21.0	6.3	2.5	VUS	c.352C>T (p.R118C)	NA
FD #2	GLA	4.4	12.0	3.3	1.1	VUS	c.352C>T (p.R118C)	NA
FD #3	GLA	7.5	15.0	3.6	1.8	VUS	c.352C>T (p.R118C)	NA
FD #4	GLA	5.8	0.0	0.2	0.0	P	c.1023A>C (p.E341D)	NA
FD #5	GLA	7.0	19.0 *	4.7	1.7	VUS	c.419A>C (p.K140T)	NA
FD #6	GLA	10.6 *	10.0 *	5.9 *	2.0	VUS	c.352C>T (p.R118C)	NA
FD #7	GLA	5.5	3.0	0.7	0.0	P	c.644A>G (p.N215S)	NA
FD #8	GLA	5.6	21.0	4.8	2.5	VUS	c.352C>T (p.R118C)	NA
FD #9	GLA	7.1	9.0	2.8	0.6	LP	c.197A>G (p.E66G)	NA
FD #10	GLA	3.9	7.0	1.5	0.0	LP	c.1088G>A (p.R363H)	NA
FD #11	GLA	6.1	6.0	2.3	0.7	LP	c.197A>G (p.E66G)	NA
FD #12	GLA	4.3	8.0	0.5	0.0	LP	c.1088G>A (p.R363H)	NA
FD #13	GLA	5.5	5.0	2.4 *	0.6	VUS	c.70T>A (p.W24R), c.1255A>G (p.N419D)	NA
FD #14	GLA	5.1	15.0	6.7 *	2.7	VUS	c.352C>T (p.R118C)	NA
FD #15	GLA	3.8	1.0	0.8	0.0	LP	c.593T>C (p.I198T)	NA
FD #16	GLA	5.5	0.0	1.0	0.0	LP	c.593T>C (p.I198T)	NA
FD #17	GLA	7.7	15.0	4.8	1.4	VUS	c.352C>T (p.R118C)	NA
FD #18	GLA	3.6	9.0	3.7	1.0	VUS	c.473C>A (p.T158N)	NA
FD #19	GLA	9.8	3.0	3.0	0.5	P	c.124A>C (p.M42L)	NA
FD #20	GLA	6.7	7.0	3.9	0.9	VUS	c.313A>G (p.R105G)	NA
FD #21	GLA	6.7	9.0	4.5	1.2	VUS	c.352C>T (p.R118C)	NA
FD #22	GLA	6.6	16.0	6.3 *	1.0	VUS	c.419A>C (p.K140T)	NA
FD #23	GLA	7.0	6.0	4.6	1.1	P	c.639+919G>A	NA
FD #24	GLA	10.0 *	17.0 *	4.6 *	1.1	VUS	c.122C>G (p.T41S)	NA
FD #25	GLA	6.8 *	22.0 *	5.9 *	1.3	PD	c.427G>A (p.A143T)	NA
FD #26	GLA	11.2 *	22.0 *	5.7 *	1.3	PD	c.427G>A (p.A143T)	NA
FD #27	GLA	9.8 *	22.0 *	4.8 *	1.6	PD	c.427G>A (p.A143T)	NA
FD #28	GLA	6.2 *	27.0 *	3.4 *	0.7	PD	c.427G>A (p.A143T)	NA
FD #29	GLA	7.2 *	19.0 *	3.1 *	0.7	PD	c.427G>A (p.A143T)	NA
FD #30	GLA	11.4 *	15.0 *	3.9 *	1.1	PD	c.427G>A (p.A143T)	NA
FD #31	GLA	6.6 *	19.0 *	4.7 *	1.4	PD	c.427G>A (p.A143T)	NA
FD #32	GLA	5.4 *	20.0 *	4.6 *	1.2	PD	c.427G>A (p.A143T)	NA
FD #33	GLA	4.2 *	13.0 *	2.8 *	1.4	PD	c.427G>A (p.A143T)	NA
FD #34	GLA	6.5 *	15.0 *	4.1 *	1.4	PD	c.427G>A (p.A143T)	NA
FD #35	GLA	4.9 *	10.0 *	2.9 *	0.7	PD	c.427G>A (p.A143T)	NA
FD #36	GLA	6.4 *	10.0 *	4.0 *	1.8	PD	c.427G>A (p.A143T)	NA
FD #37	GLA	4.6 *	13.0 *	4.0 *	0.5	PD	c.427G>A (p.A143T)	NA
FD #38	GLA	3.7 *	16.0 *	3.2 *	0.9	PD	c.427G>A (p.A143T)	NA
FD #39	GLA	8.8 *	16.0 *	5.2 *	0.9	PD	c.427G>A (p.A143T)	NA
FD #40	GLA	5.6 *	7.0 *	3.1 *	0.6	PD	c.427G>A (p.A143T)	NA
FD #41	GLA	6.0 *	13.0 *	3.4 *	0.6	PD	c.427G>A (p.A143T)	NA
FD #42	GLA	5.5 *	11.0 *	3.8 *	0.6	PD	c.427G>A (p.A143T)	NA
FD #43	GLA	6.5 *	14.0 *	6.3 *	1.3	PD	c.427G>A (p.A143T)	NA
FD #44	GLA	6.4 *	19.0 *	4.4 *	1.1	PD	c.427G>A (p.A143T)	NA
FD #45	GLA	4.1 *	18.0 *	2.1 *	0.3	PD	c.427G>A (p.A143T)	NA
FD #46	GLA	7.6 *	14.0 *	4.8 *	0.9	PD	c.427G>A (p.A143T)	NA
FD het #1	GLA	7.4 *	18.0	7.2 *	2.2	P/nd	c.870G>GC,p.M290MI	-
FD het #2	GLA	9.5*	11.0	4.2	0.7	P/nd	c.870G>A,p.M290I	-
FD het #3	GLA	7.5	9.0	5.5	1.6	P/B	c.937G>T,p.D313Y	c.1000-22C>T
FD het #4	GLA	8.0 *	16.0 *	6.5 *	2.0	PD/nd	c.427G>A (p.A143T)	-
GD #1	GBA	2.4	5.0*	0.8	0	P/P	c.1226A>G (p.N409S)	c.1226A>G (p.N409S)
GD #2	GBA	1.5	0	0	0	P/P	c.680A>G (p.N227S)	c.680A>G (p.N227S)
MPS I	IDUA	2.9	0	0.2	0	P/P	c.1205G>A (p.W402X)	c.46_57del (p.(Ser16_Ala19del))
PD #1	GAA	4.3	4.0*	1.6	0.8	P/VUS	c.-32-13T>G	c.1909C>A (p.L637M)
PD #2	GAA	5.3	0	1.7	1.1	LP/VUS	c.1292_1295dup p.(Gln433Alafs *74)	c.1019A>G (p.Y340C)
PD #3	GAA	3.8 *	0	0.5	0	P/P	c.-32-13T>G	[c.752C>T (p.S251L); c.761C>T (p.S254L)]
PD #4	GAA	4.4	0	2.5	2.6	VUS/VUS	c.257C>G (p.P86R)	c.1306C>T (p.R436W)
PD #5	GAA	5.4	0	2.4	0	P/P	c.752C>T (p.S251L)	c.761C>T (p.S254L)
PD #6	GAA	8.8	0	2.2	1.5	P/P	c.752C>T (p.S251L)	c.761C>T (p.S254L)

* Case resolved as not affected by CLIR for indicated 1st tier testing platform; ^1^ fluorometric enzyme assay (cutoffs: GLA 1.2 µmol/L/h; GBA 1.2 µmol/L/h; IDUA 1.0 µmol/L/h; GAA 2.5 µmol/L/h) [15,16,17,18]; **^2^** according to guidelines by Richards et al. [23]. FD, Fabry disease; GD, Gaucher disease; MPS I, mucopolysaccharidosis type I; PD, Pompe disease; P, pathogenic; LP, likely pathogenic; VUS, variant of uncertain significance; B, benign; PD, partial deficiency; NA, not applicable; nd, no 2nd variant (P, LP, or VUS) detected.

**Table 4:** Comparison of screening results without and with CLIR for Fabry disease (male patients and female carriers, excl. p.A143T variant) using different assay platforms and molecular genetic 2nd tier testing. The basis for this comparison is the population used for CLIR analysis (*n* = 87,321) and shows that the application of CLIR reduces the need for 2nd tier tests and improves the screening performance, except for the immunocapture and FIA-MS/MS methods where it leads to lower sensitivity.

Test Platform	Immunocapture + 2TT ^5^	Immunocapture + CLIR Tools	DMF + 2TT ^5^	DMF + CLIR Tools	MS/MS + 2TT ^5^	MS/MS + CLIR Tools
TP	27	24	22	22	23	21
Sensitivity ^1^	100%	92%	82%	82%	85%	78%
FP ^2^	74	2	74	39	74	31
Reduction in need for 2TT	-	97%	-	47%	-	58%
FPR ^3^	0.085%	0.002%	0.085%	0.045%	0.085%	0.036%
PPV ^4^	27%	92%	23%	36%	30%	40%
FN	0	3	5	5	4	6

^1^ Sensitivity calculated as true positive cases/(true positive cases + false negative cases) [TP/(TP + FN)]; ^2^ FP, false positive cases based on the first DBS sample, includes non-carriers, carriers, and cases with genotypes leading to pseudodeficient enzyme activity. ^3^ FPR, false positive rate calculated as false positive cases/(false positive cases + true negative cases) [FP/(FP + TN)]. ^4^ PPV, positive predictive value calculated as TP/(TP + FP). ^5^ 2TT, 2nd tier test by molecular genetic analysis of *GLA*; MS/MS, flow injection analysis tandem mass spectrometry.

**Table 5:** Comparison of screening results without and with CLIR for Gaucher disease using different assay platforms and 2nd tier testing. The basis for this comparison is the population used for CLIR analysis (*n* = 87,321) and shows that the application of CLIR reduces the need for 2nd tier tests and improves the screening performance.

Test Platform	Immunocapture + 2TT	Immunocapture + CLIR Tools	DMF + 2TT	DMF + CLIR Tools	MS/MS + 2TT	MS/MS + CLIR Tools
TP	1	1	2	2	2	2
Sensitivity ^1^	50%	50%	100%	100%	100%	100%
FP ^2^	107	1	107	13	107	47
Reduction in need for 2TT	-	99%	-	88%	-	56%
FPR ^3^	0.123%	0.001%	0.123%	0.015%	0.123%	0.056%
PPV ^4^	0.9%	4.1%	1.8%	13.3%	1.8%	50.0%
FN	1	1	0	0	0	0

^1^ Sensitivity calculated as true positive cases/(true positive cases + false negative cases) [TP/(TP + FN)]; ^2^ FP, false positive cases based on the first DBS sample, includes non-carriers, carriers, and cases with genotypes leading to pseudodeficient enzyme activity. ^3^ FPR, false positive rate calculated as false positive cases/(false positive cases + true negative cases) [FP/(FP + TN)]. ^4^ PPV, positive predictive value calculated as TP/(TP + FP). 2TT, 2nd tier test [17]; DMF, digital microfluidics; MS/MS, flow injection analysis tandem mass spectrometry.

**Table 6:** Comparison of screening results without and with CLIR for mucopolysaccharidosis type I using different assay platforms and 2nd tier testing. The basis for this comparison is the population used for CLIR analysis (*n* = 87,321) and shows that the application of CLIR reduces the need for 2nd tier tests and improves the screening performance.

Test Platform	Immunocapture + 2TT	Immunocapture + CLIR Tools	DMF + 2TT	DMF + CLIR Tools	MS/MS + 2TT	MS/MS + CLIR Tools
TP	1	1	1	1	1	1
Sensitivity ^1^	100%	100%	100%	100%	100%	100%
FP ^2^	159	15	159	17	159	5
Reduction in need for 2TT	-	91%	-	89%	-	97%
FPR ^3^	0.183%	0.017%	0.183%	0.020%	0.183%	0.006%
PPV ^4^	0.6%	6.3%	0.6%	5.6%	0.6%	16.7%
FN	0	0	0	0	0	0

^1^ Sensitivity calculated as true positive cases/(true positive cases + false negative cases) [TP/(TP + FN)]; ^2^ FP, false positive cases based on the first DBS sample, includes non-carriers, carriers, and cases with genotypes leading to pseudodeficient enzyme activity. ^3^ FPR, false positive rate calculated as false positive cases/(false positive cases + true negative cases) [FP/(FP + TN)]. ^4^ PPV, positive predictive value calculated as TP/(TP + FP). 2TT, 2nd tier test [16]; DMF, digital microfluidics; MS/MS, flow injection analysis tandem mass spectrometry.

**Table 7:** Comparison of screening results without and with CLIR for Pompe disease using different assay platforms and 2nd tier testing. The basis for this comparison is the population used for CLIR analysis (*n* = 87,321) and shows that the application of CLIR reduces the need for 2nd tier tests and improves the screening performance, except for the immunocapture method, where it leads to lower sensitivity.

Test Platform	Immunocapture + 2TT	Immunocapture + CLIR Tools	DMF + 2TT	DMF + CLIR Tools	MS/MS + 2TT	MS/MS + CLIR Tools
TP	6	5	5	5	6	6
Sensitivity ^1^	100%	83%	83%	83%	100%	100%
FP ^2^	99	7	99	65	99	5
Reduction in need for 2TT	-	93%	-	32%	-	95%
FPR ^3^	0.109%	0.008%	0.109%	0.075%	0.109%	0.006%
PPV ^4^	5.9%	41.7%	4.8%	7.1%	5.9%	45.5%
FN	0	1	1	1	0	0

^1^ Sensitivity calculated as true positive cases/(true positive cases + false negative cases) [TP/(TP + FN)]; ^2^ FP, false positive cases based on the first DBS sample, includes non-carriers, carriers, and cases with genotypes leading to pseudodeficient enzyme activity. ^3^ FPR, false positive rate calculated as false positive cases/(false positive cases + true negative cases) [FP/(FP + TN)]. ^4^ PPV, positive predictive value calculated as TP/(TP + FP). 2TT, 2nd tier test [18]; DMF, digital microfluidics; MS/MS, flow injection analysis tandem mass spectrometry.

## Supplementary Materials

Supplementary materials can be found at https://www.mdpi.com/2409-515X/6/2/44/s1.
