# Supplementary material for: A Comparative Effectiveness Study of Newborn Screening Methods for Four Lysosomal Storage Disorders
Source: Int J Neonatal Screen. 2020 May 30;6(2):44. doi: 10.3390/ijns6020044 (PMC7423013; doi:10.3390/ijns6020044)
Supplement: Supplementary file 1 [file IJNS-06-00044-s001.pdf]

International Journal of
Neonatal Screening

MDPI

Article

A Comparative Effectiveness Study of Newborn Screening Methods for Four Lysosomal Storage Disorders †

Karen A. Sanders [1], Dimitar K. Gavrilov [1,2], Devin Oglesbee [1,2], Kimiyo M. Raymond [1,2], Silvia Tortorelli [1,2], John J. Hopwood [3], Fred Lorey [4], Ramanath Majumdar [1], Charles A. Kroll [1], Amber M. McDonald [1], Jean M. Lacey [1], Coleman T. Turgeon [1], Justin N. Tucker [3], Hao Tang [4], Robert Currier [4,5], Grazia Isaya [6], Piero Rinaldo [1,2,6] and Dietrich Matern [1,2,6,*]

1 Biochemical Genetics Laboratory, Department of Laboratory Medicine and Pathology, Mayo Clinic, Rochester, MN 55905, USA; sanders.karen@mayo.edu (K.A.S.); gavrilov.dimitar@mayo.edu (D.K.G.); oglesbee.devin@mayo.edu (D.O.); raymond.kimiyo@mayo.edu (K.M.R.); tortorelli.silvia@mayo.edu (S.T.); Majumdar.Ramanath@mayo.edu (R.M.); kroll.charles@mayo.edu (C.A.K.); mcdonald.amberm@mayo.edu (A.M.M.); lacey.jean@mayo.edu (J.M.L.); turgeon.coleman@mayo.edu (C.T.T.); rinaldo@mayo.edu (P.R.)

2 Department of Clinical Genomics, Mayo Clinic, Rochester, MN 55905, USA

3 Lysosomal Diseases Research Unit, South Australian Health and Medical Research Institute, Adelaide 5000, Australia; john.hopwood@sahmri.com (J.J.H.); jntucker@gmail.com (J.N.T.)

4 Genetic Disease Screening Program, California Department of Public Health, Richmond, CA 94804, USA; fred_lorey@sbcglobal.net (F.L.); hao.tang@cdph.ca.gov (H.T.); robert.currier@ucsf.edu (R.C.)

5 Department of Pediatrics, University of California, San Francisco, CA 94143, USA

6 Department of Pediatric and Adolescent Medicine, Mayo Clinic, Rochester, MN 55905, USA; gisaya01@gmail.com

* Correspondence: matern@mayo.edu

† This paper is dedicated to the memory of Sue Rosenau, co-founder of the Legacy of Angels Foundation, who was a passionate supporter of research into the diagnosis and treatment of lysosomal disorders and cystic fibrosis, including this study.

Received: 4 April 2020; Accepted: 27 May 2020; Published: 30 May 2020

Table S1. Comparison of screening results for Fabry disease using different assay platforms and molecular genetic testing as employed in this study. Relevant results from other newborn screening programs are also included for additional comparison.

**Country/State**	*This study*	*This study*	**MO [1]**	*This study*	**Taiwan [2]**	**NY [3]**	**IL [4]**
**Test Platform**	*Immunocapture + GLA sequencing*	*DMF + GLA sequencing*	DMF	*FIA-MS/MS + GLA sequencing*	FIA-MS/MS	FIA-MS/MS	LC-MS/MS
**# of newborns**	*89,508*	*89,508*	308,000	*89,508*	191,767	65,605	219,793
**TP (excl. p.A143T)**	*50 (27)*	*41 (22)*	94	*44 (23)*	64	7	26
**FP[1] (excl. p.A143T)**	*53 (76)*	*53 (76)*	85	*53 (76)*	315	24	65
**FPR[2] (excl. p.A143T)**	*0.059% (0.085%)*	*0.059% (0.085%)*	0.030%	*0.059% (0.085%)*	0.16%	0.037%	0.03%
**PPV[3] (excl. p.A143T)**	*48.5% (26.2%)*	*43.6% (22.4%)*	53%	*45.4% (23.2%)*	17%	22.6%	28.6%
**FN (excl. p.A143T)**	*0 (0)*	*9 (5)*	-	*6 (4)*	-	-	-
**Incidence (excl. p.A143T): 1 in**	*1,790 (3,315)*	*1,790 (3,315)*	3,277	*1,790 (3,315)*	2,996	9,372	8,454

[1]FP, false positive cases of either sex based on the first DBS sample. [2]FPR, false positive rate calculated as FP/(FP + true negative cases). [3]PPV, positive predictive value calculated as TP/(TP + FP). FIA-MS/MS, flow injection analysis tandem mass spectrometry. LC-MS/MS, liquid chromatography MS/MS.

Table S2. Comparison of screening results for Gaucher disease using different assay platforms and 2nd tier testing as employed in this study. Relevant results from other newborn screening programs are also included for additional comparison.

**Country/State**	*This study*	*This study*	**MO [1]**	*This study*	**Taiwan [2]**	**NY [3]**	**IL [4]**
**Test Platform**	*Immunocapture + 2TT*	*DMF + 2TT*	DMF	*FIA-MS/MS + 2TT*	FIA-MS/MS	FIA-MS/MS	LC-MS/MS
**# of newborns**	*89,508*	*89,508*	308,000	*89,508*	101,134	65,605	219,793
**TP**	*1*	*2*	5	*2*	1	15	5
**FP[1]**	*111*	*111*	32	*111*	140	2	91
**FPR[2]**	*0.124%*	*0.124%*	0.010%	*0.124%*	0.138%	0.003%	0.041%
**PPV[3]**	*0.9%*	*1.77%*	13.5%	*1.77%*	0.7%	88.2%	5.2%
**FN**	*1*	*0*	-	*0*	-	-	-
**Incidence: 1 in**	*44,754*	*44,754*	61,600	*44,754*	101,134	4,374	43,959

[1]FP, false positive cases based on the first DBS sample, includes non-carriers, carriers, and cases with genotypes leading to pseudodeficient enzyme activity. [2]FPR, false positive rate calculated as FP/(FP + true negative cases). [3]PPV, positive predictive value calculated as TP/(TP + FP). 2TT, 2nd tier test; FIA-MS/MS, flow injection analysis tandem mass spectrometry. LC-MS/MS, liquid chromatography MS/MS.

Table S3. Comparison of screening results for Mucopolysaccharidosis type I using different assay platforms and 2nd tier testing as employed in this study. Relevant results from other newborn screening programs are also included for additional comparison.

**Country/State**	***This study***	***This study***	**MO [1]**	***This study***	**Taiwan [5]**	**NY [3]**	**IL [4]**
**Test Platform**	*Immunocapture + 2TT*	*DMF + 2TT*	DMF	*FIA-MS/MS + 2TT*	FIA-MS/MS	FIA-MS/MS	LC-MS/MS
**# of newborns**	*89,508*	*89,508*	308,000	*89,508*	130,237	35,816	219,793
**TP**	*1*	*1*	2	*1*	5[4]	0	1
**FP[1]**	*160*	*160*	126	*160*	115	13	126
**FPR[2]**	*0.178%*	*0.178%*	0.041%	*0.178%*	0.088%	0.036%	0.057%
**PPV[3]**	*0.6%*	*0.6%*	1.6%	*0.6%*	3.4%	-%	0.8%
**FN**	*0*	*0*	-	*0*	-	-	-
**Incidence: 1 in**	*89,508*	*89,508*	154,000	*89,508*	43,412[5]	>35,000	219,793

[1]FP, false positive cases based on the first DBS sample, includes non-carriers, carriers, and cases with genotypes leading to pseudodeficient enzyme activity. [2]FPR, false positive rate calculated as FP/(FP + true negative cases). [3]PPV, positive predictive value calculated as TP/(TP + FP). [4]TP cases include two pairs of siblings; [5]calculated based on three affected families. 2TT, 2nd tier test; FIA-MS/MS, flow injection analysis tandem mass spectrometry. LC-MS/MS, liquid chromatography MS/MS.

Table S4. Comparison of screening results for Pompe disease using different assay platforms and 2nd tier testing as employed in this study. Relevant results from other newborn screening programs are also included for additional comparison.

**Country/State**	*This study*	*This study*	**MO [6]**	*This study*	**Taiwan [2]**	**CA [7]**	**NY [3]**	**IL [8]**
**Test Platform**	*Immunocapture + 2TT*	*DMF + 2TT*	DMF	*FIA-MS/MS[4] + 2TT*	FIA-MS/MS	FIA-MS/MS + 2TT[5]	FIA-MS/MS	LC-MS/MS
**# of newborns**	*89,508*	*89,508*	467,000	*89,508*	191,786	453,152	38,816	684,290
**TP**	*6*	*5*	46	*6*	16	18	1	29
**FP[1]**	*99*	*99*	223	*99*	858	70	5	368
**FPR[2]**	*0.111%*	*0.111%*	0.048%	*0.111%*	0.447%	0.015%	0.028%	0.054%
**PPV[3]**	*5.7%*	*4.8%*	17.1%	*5.7%*	1.83%	20.45%	16.7%	7.3%
**FN**	*0*	*1*	-	*0*	-	-	-	-
**Incidence: 1 in**	*14,918*	*14,918*	10,152	*14,918*	11,987	25,175	38,816	23,596

[1]FP, false positive cases based on the first DBS sample, includes non-carriers, carriers, and cases with genotypes leading to pseudodeficient enzyme activity. [2]FPR, false positive rate calculated as FP/(FP + true negative cases). [3]PPV, positive predictive value calculated as TP/(TP + FP). [5]Molecular genetic analysis of *GAA*; 2TT, 2nd tier test; FIA-MS/MS, flow injection analysis tandem mass spectrometry. LC-MS/MS, liquid chromatography MS/MS.

References

1. Hopkins, P.V.; Klug, T.; Vermette, L.; Raburn-Miller, J.; Kiesling, J.; Rogers, S. Incidence of 4 Lysosomal Storage Disorders From 4 Years of Newborn Screening. *JAMA Pediatr* **2018**, *172*, 696-697, doi:10.1001/jamapediatrics.2018.0263.
2. Liao, H.C.; Chiang, C.C.; Niu, D.M.; Wang, C.H.; Kao, S.M.; Tsai, F.J.; Huang, Y.H.; Liu, H.C.; Huang, C.K.; Gao, H.J., et al. Detecting multiple lysosomal storage diseases by tandem mass spectrometry--a national newborn screening program in Taiwan. *Clin Chim Acta* **2014**, *431*, 80-86, doi:10.1016/j.cca.2014.01.030.
3. Wasserstein, M.P.; Caggana, M.; Bailey, S.M.; Desnick, R.J.; Edelmann, L.; Estrella, L.; Holzman, I.; Kelly, N.R.; Kornreich, R.; Kupchik, S.G., et al. The New York pilot newborn screening program for lysosomal storage diseases: Report of the First 65,000 Infants. *Genet Med* **2019**, *21*, 631-640, doi:10.1038/s41436-018-0129-y.
4. Burton, B.K.; Charrow, J.; Hoganson, G.E.; Waggoner, D.; Tinkle, B.; Braddock, S.R.; Schneider, M.; Grange, D.K.; Nash, C.; Shryock, H., et al. Newborn Screening for Lysosomal Storage Disorders in Illinois: The Initial 15-Month Experience. *J Pediatr* **2017**, *190*, 130-135, doi:10.1016/j.jpeds.2017.06.048.
5. Chan, M.J.; Liao, H.C.; Gelb, M.H.; Chuang, C.K.; Liu, M.Y.; Chen, H.J.; Kao, S.M.; Lin, H.Y.; Huang, Y.H.; Kumar, A.B., et al. Taiwan National Newborn Screening Program by Tandem Mass Spectrometry for Mucopolysaccharidoses Types I, II, and VI. *J Pediatr* **2019**, *205*, 176-182, doi:10.1016/j.jpeds.2018.09.063.
6. Klug, T.L.; Swartz, L.B.; Washburn, J.; Brannen, C.; Kiesling, J.L. Lessons Learned from Pompe Disease Newborn Screening and Follow-up. *Int. J. Neonatal Screen.* **2020**, *6*, 11.
7. Tang, H.; Feuchtbaum, L.; Sciortino, S.; Matteson, J.; Mathur, D.; Bishop, T.; Olney, R.S. The First Year Experience of Newborn Screening for Pompe Disease in California. *Int. J. Neonatal Screen.* **2020**, *6*, 9.
8. Burton, B.K.; Charrow, J.; Hoganson, G.E.; Fleischer, J.; Grange, D.K.; Braddock, S.R.; Hitchins, L.; Hickey, R.; Christensen, K.M.; Groeppner, D., et al. Newborn Screening for Pompe Disease in Illinois: Experience with 684,290 Infants. *Int. J. Neonatal Screen.* **2020**, *6*, 4.
